# *Snx27* Deletion Promotes Recovery From Spinal Cord Injury by Neuroprotection and Reduces Macrophage/Microglia Proliferation

**DOI:** 10.3389/fneur.2018.01059

**Published:** 2018-12-13

**Authors:** Yuzhe Zeng, Nawen Wang, Tiantian Guo, Qiuyang Zheng, Shuang Wang, Songsong Wu, Xi Li, Jin Wu, Zhida Chen, Huaxi Xu, Xin Wang, Bin Lin

**Affiliations:** ^1^Department of Orthopaedics, The Affiliated Southeast Hospital of Xiamen University, Orthopaedic Center of People's Liberation Army, Zhangzhou, China; ^2^Fujian Provincial Key Laboratory of Neurodegenerative Disease and Aging Research, Institute of Neuroscience, College of Medicine, Collaborative Innovation Center for Brain Science, Xiamen University, Xiamen, China; ^3^Neuroscience Initiative, Sanford Burnham Prebys Medical Discovery Institute, La Jolla, CA, United States; ^4^State Key Laboratory of Cellular Stress Biology, Xiamen University, Xiamen, China

**Keywords:** sorting nexin 27, neuroprotection, macrophage/microglia, functional recovery, spinal cord injury

## Abstract

Sorting nexin 27 (SNX27) is an endosome-associated cargo adaptor that is involved in various pathologies and development of neurological diseases. However, the role of SNX27 in spinal cord injury (SCI) remains unclear. In this study, we found that SNX27 was up-regulated in injured mice spinal cords by western blot and immunofluorescence. A comparative analysis of Basso mouse scale (BMS), footprint test and corticospinal tract (CST) tracing in *Snx27*^+/+^ and *Snx27*^+/−^ mice revealed that haploinsufficiency of SNX27 ameliorated the clinical symptoms of SCI. Based on the results of western blot and immunofluorescence, mechanistically, we found that SNX27 deficiency suppresses apoptotic caspase-3 induced neuronal death. In addition, SNX27 haploinsufficiency lowers the infiltration and activation of macrophage/microglia by suppressing their proliferation at the SCI lesion site. Together, these results suggest that down-regulation of SNX27 is a potential therapy targeting both acute neuronal death and chronic neuroinflammation, and promoting nerve repair after SCI.

## Introduction

Spinal cord injury (SCI) is a traumatic event resulting in sensory, motor, and autonomic dysfunction and has direct impacts on the quality of life of affected individuals (1), pharmacological treatments for SCI are still very limited (2). In addition, secondary injuries (such as edema, ischemia, glial activation, neuroinflammation, and excitotoxicity) can further exacerbate the damage to the cord itself (3–5). However, the underlying mechanisms are still largely unknown, many factors could be involved in this process (6). Among these factors, excitotoxicity and neuroinflammation are two main mechanisms that cause the overall effects to the spinal cord (3).

Excitotoxicity is a kind of neuronal cell death caused by excessive activation of glutamate receptors due to increasing levels of extracellular glutamate at the lesion site. Additionally, the increased activation of NMDA receptors contributes to extensive spinal dysfunction. Previous studies show that neuronal death due to CNS injury can be decreased by down-regulation of NMDA receptors such as GluN1 and GluN2B (7, 8). Neuroinflammation responses involve inflammatory cell infiltration (such as neutrophils and macrophages), in addition to microglia activation. Together these cells release a large number of pro-inflammatory cytokines and neurotoxins leading to neuronal death, axonal interruption adjacent to the primary lesion, and hindered axonal regeneration (9–12). It has been shown that regulation of posttraumatic inflammation is required to improve functional recovery (13, 14), such as through depletion of macrophages (15) and delivery of anti-inflammatory chemicals to the spinal cord lesion site (16, 17). Therefore, SCI treatment strategies should not only focus on removal of injury factors but also need to suppress the excitotoxicity and the activation of macrophage/microglias (18–21).

Sorting nexin 27 (SNX27) is an endosome-associated cargo adaptor (22) that is involved in the development and pathologies of neurological diseases (23). SNX27-deficiency causes cognitive impairment and contributes to the pathologies of Down syndrome by regulating glutamate receptor recycling (24) and contributes to Alzheimer disease pathologies by controlling APP processing (25, 26). Furthermore, the contributions of SNX27 in a wide range of neurological diseases have been characterized ranging from infantile myoclonic epilepsy (27) and hydrocephalus (28), to drug addiction (29), and neuropathic pain (30). Together, these findings demonstrate the vital role of SNX27 in neurological disorders and neuropathic injuries such as SCI.

Despite its well-characterized roles in various neurological disorders, the role of SNX27 in SCI remains unclear. Previous studies show that nerve ligation induces allodynia related to elevated expression of SNX27 and knock down of spinal SNX27 ameliorates allodynia induced by spinal nerve ligation (30), suggesting a potential role of SNX27 in SCI. In this study, we took advantage of a compression model which clinically resembles SCI by fracture dislocations and burst fractures (31). We found that SNX27 was up-regulated in injured mouse spinal cords. SNX27 haploinsufficiency reduced neuronal loss and cleaved caspase 3, as well as the infiltration and proliferation of macrophage/microglia. Moreover, haploinsufficiency of SNX27 improved motor function recovery and corticospinal axon regeneration in *Snx27*^+/−^ mice after SCI. These findings suggest that down regulation of SNX27 is a potential therapeutic target to overcome the two main obstacles to nerve repair after SCI.

## Materials and Methods

### Animals

*Snx27*^+/+^ and *Snx27*^+/−^ mice were generated by crossing heterozygotes in the C57BL/6 background and were bred in the Animal Center of Xiamen University. All mice used for experiments were females between the ages of 10 and 14 weeks (weight 20–25 g). Pairs of female *Snx27*^+/+^ and *Snx27*^+/−^ mice were matched by age with maximum differences of 2 weeks (24). All mice were housed in a specific pathogen-free laboratory animal room and given access to a 12 h light-dark cycle in a 18~22°C facility, with free access to food and water. *Snx27*^+/+^ and *Snx27*^+/−^ mice were randomly assigned into two groups: the sham group and SCI group, through a completely randomized digital table. Observers were blinded to the grouping and experimental design during data collection and analysis. The total number of animals has been summarized in Table 1.

**Table 1:** Summary of the total number of animals.

	**Groups**	**C57BL/6**	**SCI**		**Sham**		**Total**
			***Snx27^**+/+**^***	***Snx27^**+/−**^***	***Snx27^**+/+**^***	***Snx27^**+/−**^***	
Experiment	Behavioral assessment	0	12	12	12	12	48
	BDA tracing (followed by behavioral assessment)	0	0	0	0	0	0
	Immunohistochemistry	6	19	19	0	0	44
	Immunoblotting	20	11	11	0	0	42
Total		26	42	42	12	12	134

### Antibody

The antibodies used were as follows: rabbit anti-mouse SNX27 (1:50, Thermo Fisher Scientific, #23025), rabbit anti-mouse Glial Fibrillary Acidic Protein, GFAP (1:500, WAKO, #Z0334), rabbit anti-mouse Ionized calcium-binding adapter molecule 1, Iba1 (1:250, DAKO, #019-19741), rabbit anti-mouse cleaved-caspase3 (1:800, Cell Signaling Technology, #9664S), mouse anti-mouse NeuN (1:400, EMD Millipore, #MAB377), rabbit anti-mouse β-actin (1:5,000, proteintech, #20536-1-AP), Alexa-fluor-488-conjugated goat anti-rabbit IgG (1:500, Thermo Fisher Scientific, #11034), Alexa-fluor-546-conjugated goat anti-mouse IgG (1:500, Thermo Fisher Scientific, #11081), HRP-conjugated goat anti-rabbit IgG (H + L) (1:10,000, Thermo Fisher Scientific, #31460), and HRP-conjugated goat anti-mouse IgG (H +L) (1:10 000, Thermo Fisher Scientific, #31430).

### Compressive Injury and Surgical Procedures

The surgical procedures for SCI were described previously (32). After mice were anesthetized with ketamine (100 mg/kg, *i.p*.) and xylazine (15 mg/kg, *i.p*.), the T9 lamina was removed and a compressive injury to the spinal cord was inflicted with No. 5 Dumont forceps (Fine Science Tools) modified with a spacer making the maximal closure 0.4 mm, which was applied for 60 s. Surgeries were performed by a surgeon who was blinded to the group allocation. The incision was closed in layers. Postoperatively, 1 mL of saline solution was administered to prevent dehydration. The bladder was pressed 2 times per day until the bladder reflex was re-established. All animals were housed 3 per cage in a controlled environment on a 12/12-h dark and light cycle.

### Behavioral Assessment

#### Basso Mouse Scale (BMS)

BMS, a standardized locomotor rating scale, was used to examine the motor recovery of injured animals (33). Before surgical procedures, mice were acclimated to the open field environment for 1 week. This rating scale assesses not only limb movement, stepping, and coordination, but also trunk stability in an open field. Animals with better locomotor recovery are given a higher score. The test was performed before the surgical procedures (day−1) and on days 1, 7, 14, 21, and 28 after the injury.

#### Footprint Test (33)

For footprint analysis, the hind paws were painted with black ink to record the walking pattern across a paper runway (3 × 30 cm) during continuous locomotion 4 weeks after the injury. The stride lengths and widths were measured and analyzed only when the mice ran at a constant velocity.

The data were collected by two researchers blinded to the experiment design.

### BDA Tracing

Mice were anesthetized and placed on a rodent stereotaxic frame, and a midline incision was made to reveal the bregma. Four small holes in the skull were bored with a microdrill and biotinylated dextran amine (BDA, 10% solution in 0.1 M PBS, Invitrogen, D1956) was injected into the right motor cortex using a Hamilton syringe. Four injections of 0.5 μL were injected at a rate of 0.05 μL/min. The coordinates were as follows: 1.5 mm lateral, 0.6 mm deep, and 0.5 mm anterior; 0.0, 0.5, and 1.0 mm caudal to the bregma. The mice were sacrificed 14 days after BDA injection to visualize the corticospinal tract (CST) axons (34, 35).

### BDA-Labeled Axon Counts

BDA-labeled axons were detected by application of Streptavidin-Alexa 488 (Thermo Fisher Scientific) in the sagittal section. The number of fibers was analyzed with a confocal microscope. BDA-labeled axons were quantified between the track end and the lesion site. The number of BDA labeled axons at different distances from the lesion center were quantified. BDA labeled axons were counted from five to seven adjacent sections per animal by a person blinded to the experiment design.

### Histology

Anesthetized mice were transcardially perfused with ice-cold PBS (0.1 M; pH 7.4) followed by 4% paraformaldehyde (PFA) in PBS. The thoracic area of the spinal cord was removed and cleaved into a 1 cm segment. The tissues were post-fixed in 4% PFA overnight followed by cryoprotection in 30% sucrose for another 48 h. Then, the tissues were embedded in OCT and serially sectioned into 15 μm slices with a Leica CM1860 Cryostat.

### Immunohistochemistry

Before staining, spinal cord sections were dried at 60°C for 1 h, and then rinsed with 0.1 M PBS 3 times. After blocking in 5% goat serum, the sections were incubated with indicated primary antibodies at 4°C overnight. The next day, the slides were rinsed with 0.1 M PBS and incubated for 1 h in secondary antibodies. After rinsing with 0.1 M PBS, the sections were counterstained with DAPI and mounted with fluoromount G. Images were acquired by confocal fluorescence microscopy (Nikon Microsystems). All the measurements were made by a person blinded to this experiment design.

### Immunoblotting

Animals were sacrificed and the spinal cord tissue was quickly dissected. A segment 0.5 cm long centered at the lesion site was removed. Tissues were lysed in RIPA lysis buffer (25 mM Tris-HCl, pH 7.6, 150 mM NaCl, 1% sodium deoxycholate, 1% Nonidet P-40, 0.1% sodium dodecyl sulfate), supplemented with protease inhibitors (Roche). Equal amounts of protein lysates were subjected to SDS-polyacrylamide gel electrophoresis, transferred to a PVDF membrane (EMD Millipore), and blotted with indicated antibodies. The protein levels were quantified by Image J software by a person blinded to the experiment design and the acquired data were normalized to β-actin.

### Statistical Analysis

Statistical differences between groups were calculated with an unpaired two-tailed Student's *t*-test. Other analyses were performed using two-way analysis of variance (ANOVA) with Tukey's *post-hoc* multiple-comparison test as appropriate to the design. The variance similarity between samples was confirmed using *t*-tests. All analyses were conducted using GraphPad Prism software version 7.0. All data are presented as mean ± S.E.M.

## Results

### Expression of SNX27 Is Up-Regulated in the Injured Spinal Cord

**Figure 1:**
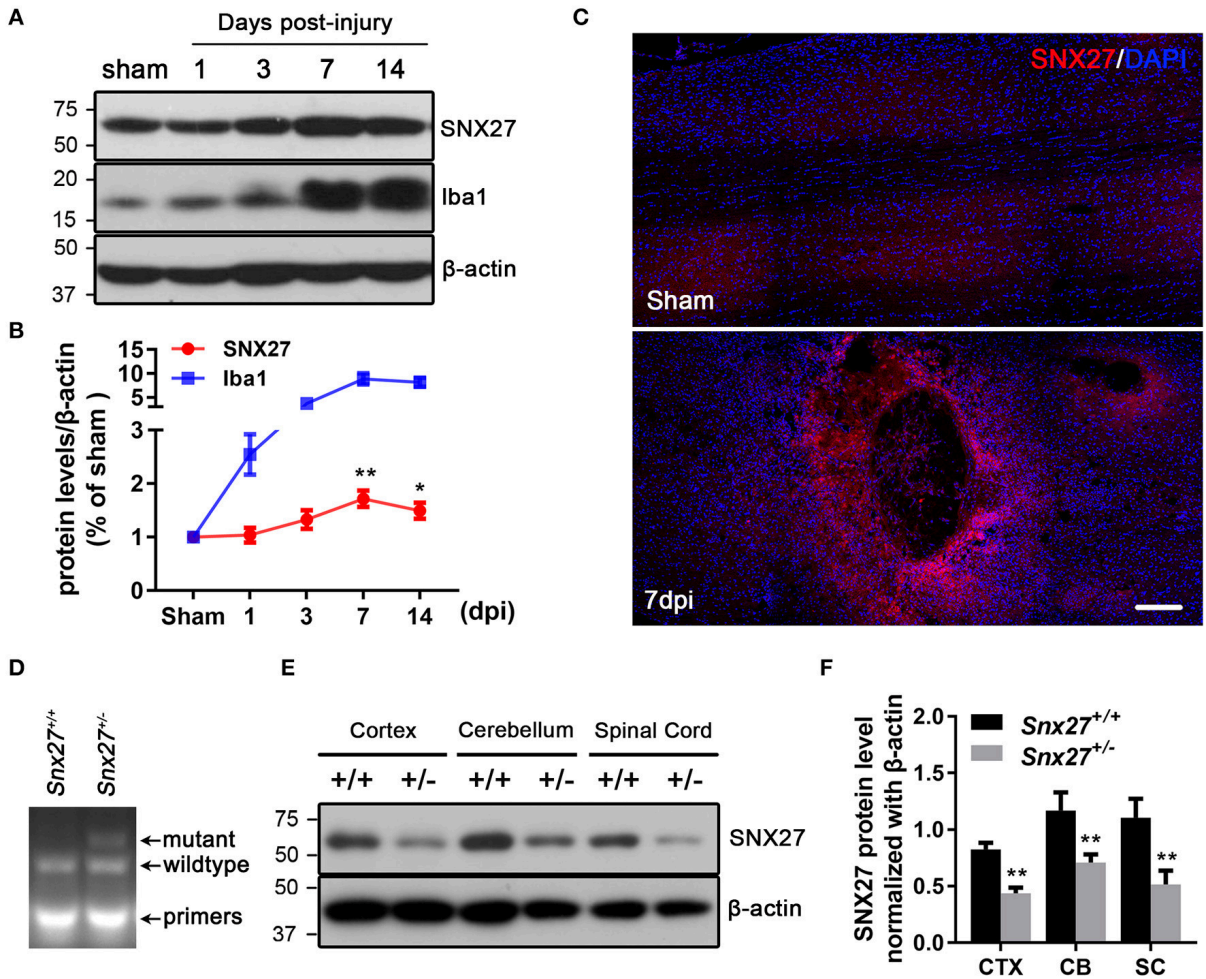
Expression of SNX27 in the injured spinal cord and the identification of *Snx27*^+/−^ mice. **(A)** Western Blot analysis of SNX27 and Iba1 levels in spinal cords from sham SCI treated mice. **(B)** Time course analysis of SNX27 expression at the SCI lesion site, *n* = 4 mice per time point. **(C)** Histological analysis of SNX27 expression in the spinal cords from sham and SCI mice 7 days post injury. Scale bar = 200 μm. **(D)** PCR genotyping of *Snx27*^+/+^ and *Snx27*^+/−^ mice. **(E,F)** Western Blot analysis of SNX27 in *Snx27*^+/+^ and *Snx27*^+/−^ mice. The results were represented as the mean ± SEM and data were evaluated by One-way ANOVA with Tukey *post-hoc* test, *p < 0.05, **p < 0.01. dpi, days post injury. CTX, Cortex; CB, Cerebellum; SC, Spinal Cord.

To assess the pathological function of SNX27 following SCI, we analyzed the expression of SNX27 in the spinal cord of sham and injured mice. Expression levels of SNX27 were significantly increased in the lesion sites of the SCI-group, but not the sham-groups at days 3, 7, and 14 after injury (Figure 1A). This up-regulation of SNX27 protein levels was detected on day 3 after SCI and reached its peak on day 7 post-injury (Figure 1B). Furthermore, immunofluorescence analysis indicated that SNX27 expression was strongly upregulated in SCI-groups compared to the sham-groups on day 7 post-injury (Figure 1C). Interestingly, the expression of SNX27 was particularly concentrated in the lesion site and was upregulated in the early stage of SCI, which suggests that SNX27 might have an impact on neuronal death and inflammation.

### SNX27 Haploinsufficiency Promotes Functional Recovery After SCI

We investigate the roles of SNX27 in the pathogenesis of SCI using *Snx27*^+/−^ mice, as homozygous knockout of SNX27 results in severe developmental retardation and early lethality in *Snx27*^−/−^ mice, making it impossible to determine whether SNX27 influences functional recovery after SCI (24). The Tail DNA products from *Snx27*^+/+^ and *Snx27*^+/−^ mice were genotyped by PCR (Figure 1D). Meanwhile, SNX27 protein expression in spinal cord, cortex and cerebellum of *Snx27*^+/+^ and *Snx27*^+/−^ mice was evaluated by western blot analysis and we found a reduction of SNX27 expression (about 50%) in *Snx27*^+/−^ mice compared to that in *Snx27*^+/+^ mice (Figures 1E,F).

**Figure 2:**
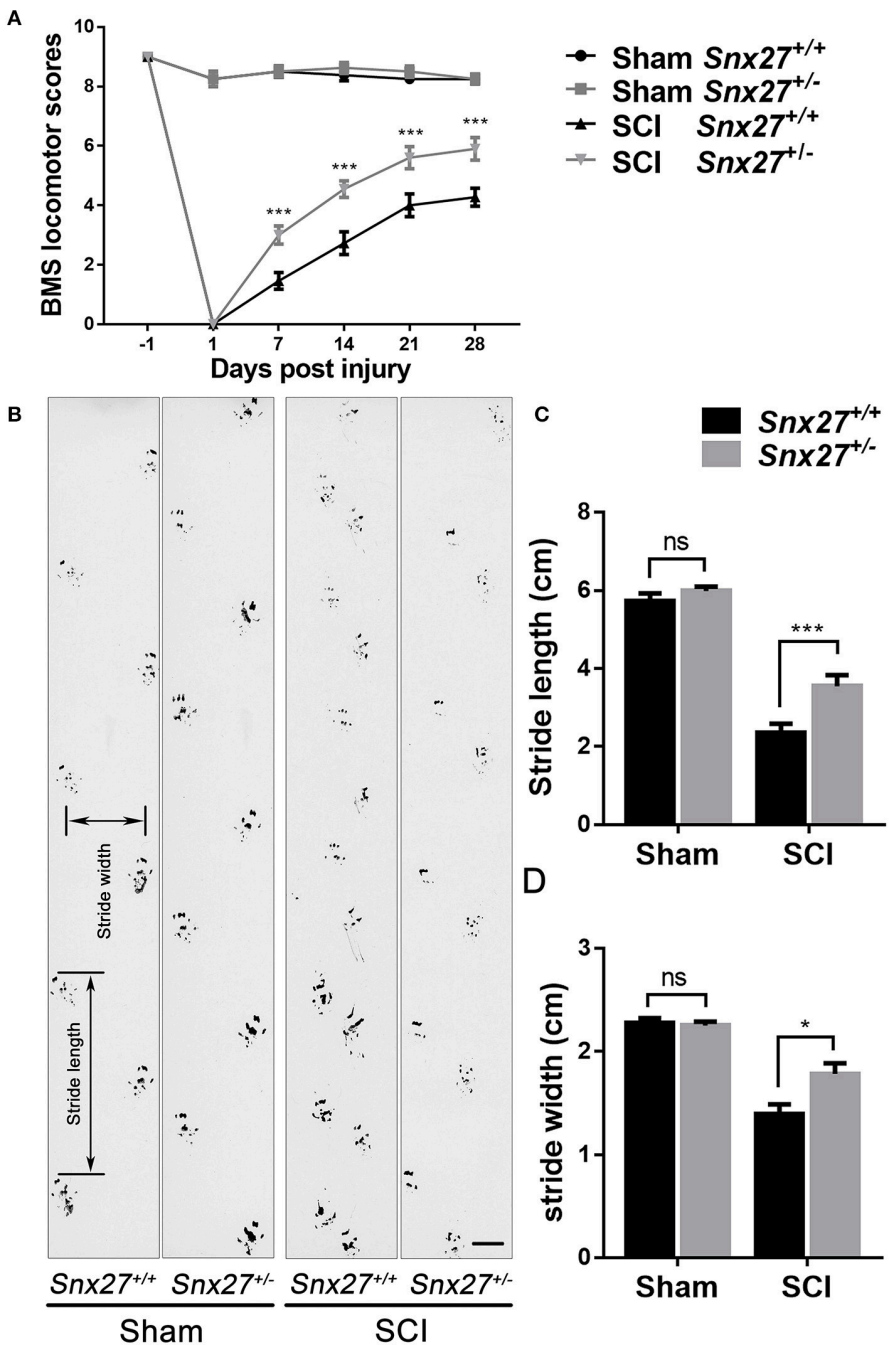
SNX27 haploinsufficiency promotes functional recovery after SCI. **(A)** Evaluating functional recovery of *Snx27*^+/+^ and *Snx27*^+/−^ mice after SCI using BMS scoring, *n* = 12. Scale bar = 1cm. **(B)** Representative images of footprint analysis 28 days post injury. **(C,D)** Quantification of stride width and stride length in the footprint analysis 28 days post injury, *n* = 12. Values are expressed as mean ± SEM, Data were analyzed using repeated measures ANOVA followed by Bonferroni's *post-hoc* test, *p < 0.05, ***p < 0.001.

To investigate the effects of SNX27 on motor recovery after SCI, footprint analysis, and open field locomotion tests were performed to objectively assess the functional improvements in *Snx27*^+/+^ and *Snx27*^+/−^ mice at week 4 after SCI. The sham groups of both *Snx27*^+/+^ and *Snx27*^+/−^ mice displayed normal functional outcomes. On day 1 post-injury, both genotypes displayed significant hind limb paralysis. However, most *Snx27*^+/−^ mice showed consistent plantar stepping and consistent coordination (BMS score: 6 or 7) on day 28 post-injury. In contrast, *Snx27*^+/+^ mice had little to no coordination and rotated paw position (BMS score: 4 or 5) although they displayed frequent or consistent plantar stepping (Figure 2A). In contrast to *Snx27*^+/+^ mice, *Snx27*^+/−^ mice exhibited longer stride length and width of the hind limb in footprint analyses at week 4 post-injury. Together, these data indicate that SNX27 is involved in functional disabilities of the spine and recovery of hind limb motor function, which was significantly improved in *Snx27*^+/−^ mice after SCI (Figures 2B–D).

### SNX27 Haploinsufficiency Increases Axon Regeneration After SCI

**Figure 3:**
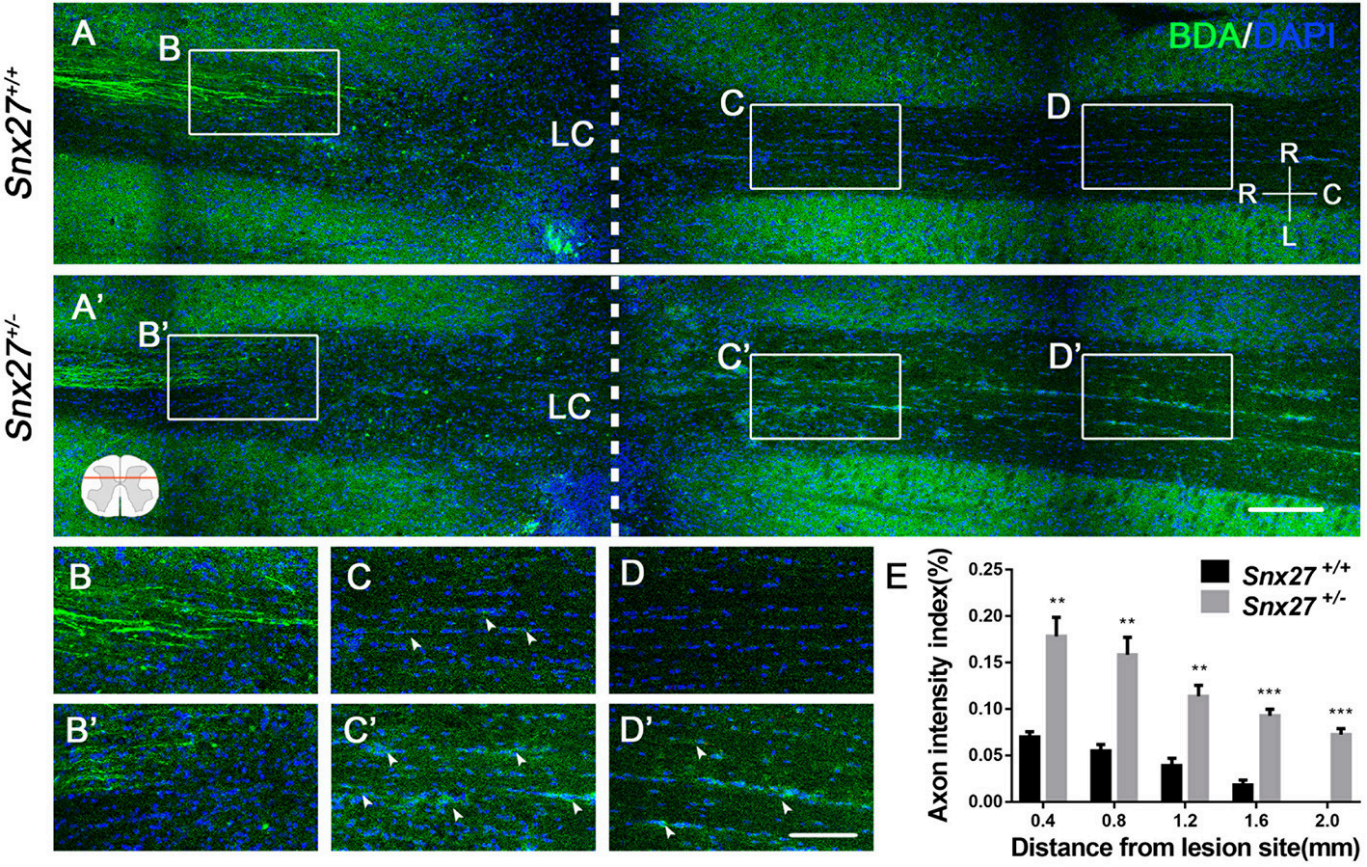
SNX27 haploinsufficiency increases axon regeneration after SCI. **(A)** Overview of BDA-labeled nerve fibers (green) in horizontal sections of *Snx27*^+/+^ mice and *Snx27*^+/−^ mice dorsal columns, ranging from rostral 1,500 μm (−1,500 μm) to caudal 2,000 μm (+2,000 μm) around the LC, Scale bar = 200 μm. R-C, Rostral–caudal; R-L, right–left. Dashed lines indicate the lesion center (LC). **(B,B′,C,C′,D,D′)** Higher magnification of the boxed areas in A. Arrowheads indicate BDA-labeled nerve fibers. Scale bar = 100 μm. **(E)** Quantification of BDA-labeled nerve fibers crossing the lesion site. *n* = 8 mice per genotype. Values are expressed as mean ± SEM and data were evaluated by One-way ANOVA with Tukey *post-hoc* test. **p < 0.01, ***p < 0.001. LC, Lesion Center.

Corticospinal tract (CST) growth is correlated with functional recovery after SCI (36). Therefore, we wondered whether SNX27 is involved in the recovery of CST after SCI. We injected biotinylated dextran amine (BDA) into the right sensorimotor cortex of mice to label the CST on day 28 post-injury and all mice were sacrificed after 2 weeks. The number of BDA^+^ nerve fibers crossing the compressive injury lesion site were quantified to evaluate functional improvement. In *Snx27*^+/+^ mice, most of the BDA^+^ nerve fibers retracted from the lesion site (Figure 3A). In contrast, *Snx27*^+/−^ mice displayed vigorous regrowth of nerve fibers (Figure 3A′), with more continuous BDA^+^ axons traversing the lesion site and growing into the distal spinal cord about 0.8 mm to the caudal lesion site (Figure 3B′,C′). Quantification of the BDA^+^ axons of CST caudal to the lesion site showed that *Snx27*^+/−^ mice had significantly more axons in the spinal cords 2 mm away from the lesion site than *Snx27*^+/+^ mice (Figure 3D). All these data imply that SNX27-deficiency might enhance corticospinal axon regeneration after SCI.

### SNX27 Haploinsufficiency Prevents Neuronal Death and Caspase-3 Activation After SCI

**Figure 4:**
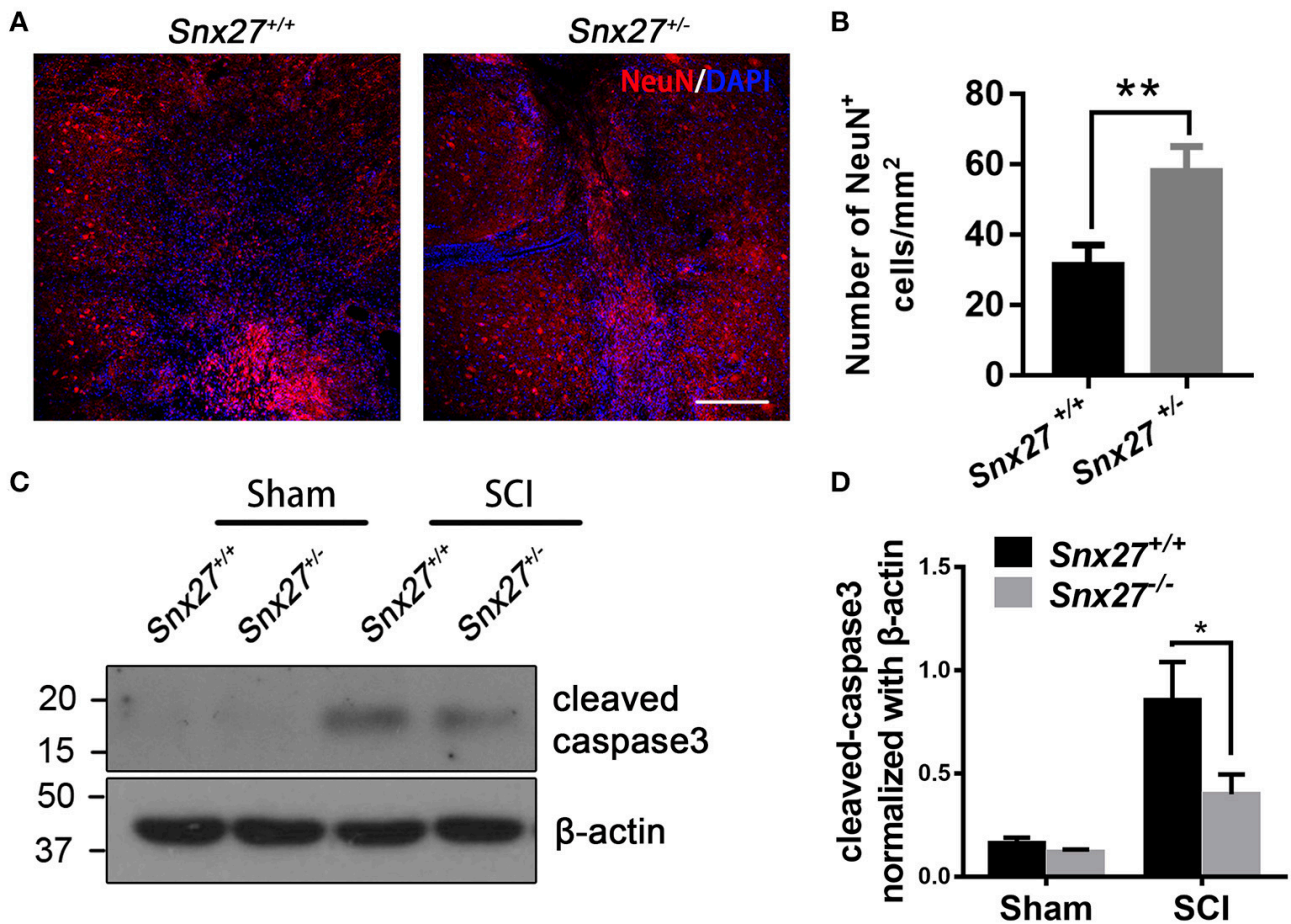
SNX27 haploinsufficiency prevents neuronal death and caspase-3 activation after SCI. **(A)** Representative images of the lesion center in the spinal cords from *Snx27*^+/+^ mice and *Snx27*^+/−^ mice 7 days post injury, Scale bar = 200 μm. **(B)** NeuN^+^ cell quantification of the lesion center in the spinal cords from *Snx27*^+/+^ mice and *Snx27*^+/−^ mice 7 days post injury, *n* = 4. **(C)** Western blot analysis of cleaved caspase-3 levels in the spinal cord of *Snx27*^+/+^ mice and *Snx27*^+/−^ mice 3 days post injury. **(D)** Quantitative analysis of cleaved caspase-3 expression in the spinal cord of *Snx27*^+/+^ mice and *Snx27*^+/−^ mice 3 days post injury, *n* = 4. Values are expressed as mean ± SEM and were evaluated by Student's independent sample *t*-test. *p < 0.05, **p < 0.01.

To determine the effects of SNX27 on neuronal survival after SCI, the number of NeuN-positive cells adjacent to the lesion site was counted on day 3 post-injury. Consistent with the behavioral data, there were more NeuN-positive cells adjacent to the lesion site in *Snx27*^+/−^ mice compared with *Snx27*^+/+^ mice (Figures 4A,B).

Caspase-3 is activated after SCI as a key execution in neuronal apoptosis (37, 38). To investigate the effect of SNX27 on apoptotic cell death after SCI, we tested for the presence of active cleaved caspase-3 after SCI in each group and found that the level of cleaved caspase-3 was indeed increased after SCI. However, cleaved caspase-3 was highly decreased in the SCI-*Snx27*^+/−^ group compared with SCI-*Snx27*^+/+^ group (Figures 4C,D). Taken together, our results indicate that SNX27 haploinsufficiency confers neuroprotection possibly via decreasing activation of caspase 3.

### SNX27 Haploinsufficiency Reduces Inflammatory Responses but Does Not Influence Gliosis After SCI

**Figure 5:**
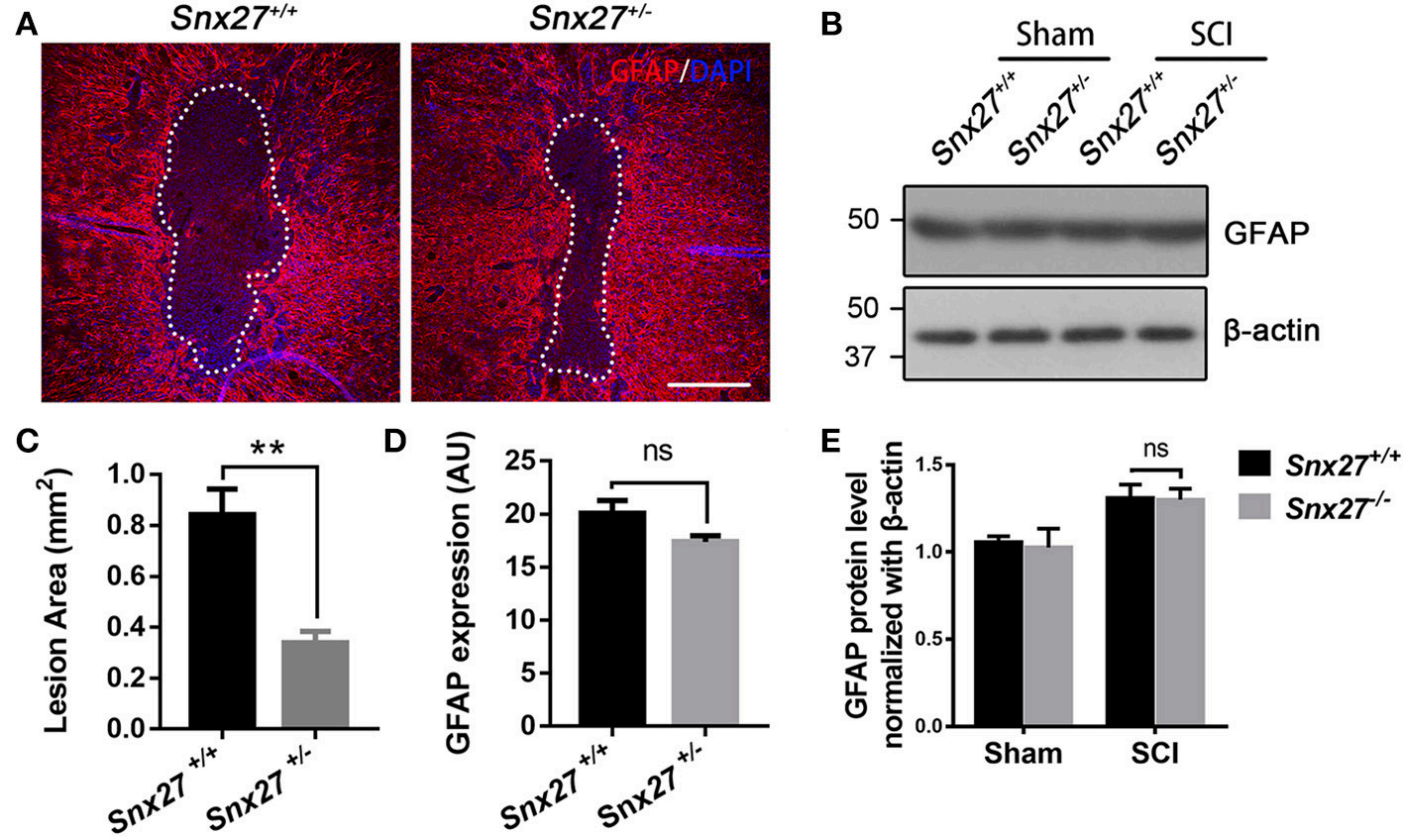
SNX27 haploinsufficiency reduces the size of lesion region without influencing gliosis after SCI. **(A)** Representative images of GFAP staining indicating the lesion region 7 days post injury, *n* = 6, Scale bar = 200 μm. Dashed lines indicate the lesion area. **(B)** Western blot analysis of GFAP in the spinal cord of *Snx27*^+/+^ mice and *Snx27*^+/−^ mice 7 days post injury. **(C,D)** Quantification of GFAP-negative area (lesion region) and GFAP protein expression, *n* = 6. **(E)** Quantitative analysis of GFAP expression, *n* = 3. Values are expressed as mean ± SEM and were evaluated by Student's independent sample *t*-test. **p < 0.01.

Increased glial fibrillary acidic protein (GFAP) expression is associated with scar formation, a secondary damage after SCI, and is an indicator of reactive gliosis. Sagittal sections were stained for GFAP to examine the pathological effects of SNX27 on lesion size and astrocyte reactivity after SCI. The lesion volume, of the GFAP-negative area, was comparable between *Snx27*^+/+^ and *Snx27*^+/−^ mice on day 7 after SCI, but the GFAP-negative area was significantly smaller in *Snx27*^+/−^ mice (Figures 5A,C). However, there was no difference in reactive astrocytes indicated by GFAP expression in the regions adjacent to the lesion sites between *Snx27*^+/+^ and *Snx27*^+/−^ mice (Figures 5B,D,E).

**Figure 6:**
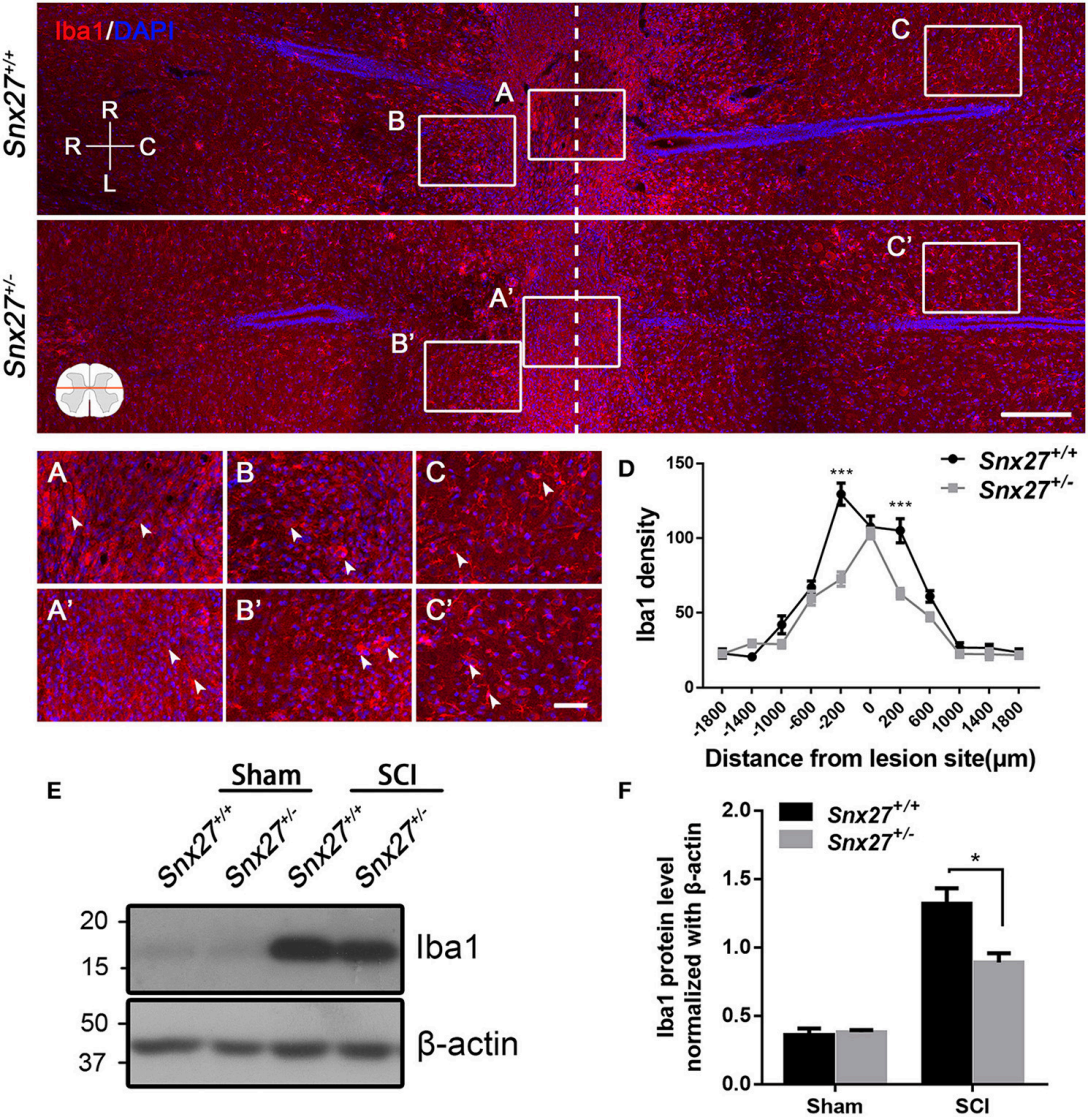
SNX27 haploinsufficiency reduces the activation of macrophage/microglia in the injured spinal cord. Top, Overview of Iba1-positive cell in horizontal sections of *Snx27*^+/+^ mice and *Snx27*^+/−^ mice dorsal spinal columns, ranging from rostral 1,500 μm (–1,500 μm) to caudal 1,500 μm (+1,500 μm) around the LC. *n* = 6. Scale bar = 200 μm. R-C, Rostral–caudal; R-L, right–left. Dashed lines indicate the lesion center. Below, Higher magnification of the different zones: the lesion centers **(A,A′)**; the injury boundary zones **(B,B′)**; the non-injury zones **(C,C′)**, Scale bar = 50 μm. Arrowheads indicate the quiescent and activated microglia. **(D)** Quantification of Iba1 density. **(E)** Western blot analysis of Iba1 expression in the spinal cords from *Snx27*^+/+^ mice and *Snx27*^+/−^ mice 7 days post injury. **(F)** Quantitative analysis of Iba1 expression, *n* = 4. Values are expressed as mean ± SEM and data were evaluated by One-way ANOVA with Tukey *post-hoc* test. *p < 0.05, ***p < 0.001.

Macrophage/microglia activation contributes to the development of secondary injury after SCI. Therefore, we analyzed the expression pattern of Iba1, a marker for macrophage/microglia activation and accumulation, in the spinal cord of SCI mouse models. Increased number of round and amoeboid-like Iba1^+^ cells (activated macrophage/microglia) have been observed in the injured boundary zone and lesion center, but the Iba1^+^ cells in the non-injury site remained ramified (quiescent macrophage/microglia). In addition, we found that *Snx27*^+/−^ mice had reduced Iba1 density in the injured boundary zone of the spinal cord compared with *Snx27*^+/+^ littermates (Figures 6A,A′,D), but had no difference in the zones of the lesion center (Figures 6B,B′) or the non-injury sites on day 7 post-injury (Figures 6C,C′). Expression of Iba1 was 5- to 6-fold higher in injured spinal cords compared with sham tissues in both groups (Figures 6E,F). However, *Snx27*^+/−^ mice had significantly reduced Iba1 upregulation than *Snx27*^+/+^ mice. These results indicate that SNX27 haploinsufficiency suppresses inflammatory responses but not scar formation after SCI.

### SNX27 Haploinsufficiency Suppresses the Proliferation of Macrophage/Microglia After SCI

**Figure 7:**
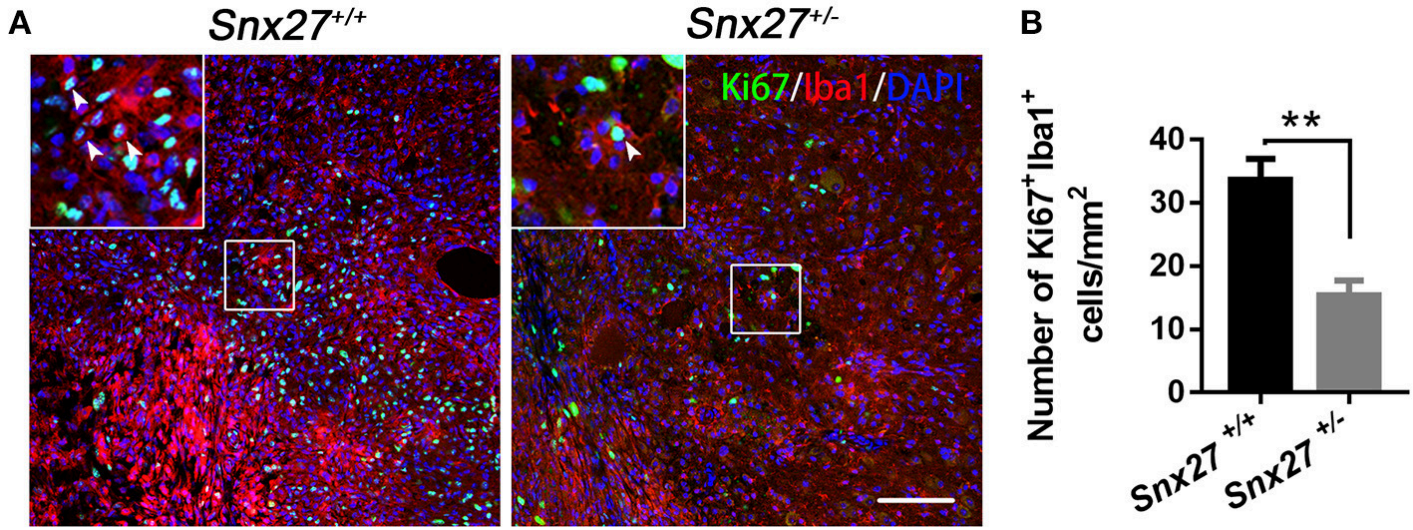
SNX27 haploinsufficiency suppresses the proliferation of macrophage/microglia after SCI. **(A)** Immunohistological analysis of horizontal spinal cord sections from *Snx27*^+/+^ mice and *Snx27*^+/−^ mice 7 days post injury using antibodies against Iba1 and Ki67 in horizontal sections. Arrowheads indicate Ki67^+^Iba1^+^ cells. **(B)** Quantification of Ki67^+^Iba1^+^ cells at the area which is ranging from rostral 400 μm (−400 μm) to caudal 400 μm (+400 μm) around the LC, *n* = 3. Values are expressed as mean ± SEM and were evaluated by Student's independent sample *t*-test. **p < 0.01.

SNX27 haploinsufficiency suppresses inflammatory responses but not scar formation after SCI, implying that SNX27 might be involved in the proliferation of microglial/macrophage cells that migrate toward and infiltrate the lesion site. Therefore, double immunostaining for Iba1 and Ki67, a cellular marker for proliferation (39), was performed in sagittal sections of the spinal cords on day 7 post-injury. In contrast to *Snx27*^+/+^ littermates, *Snx27*^+/−^ mice exhibited fewer Ki67^+^ cells in the lesion site (Figure 7A), indicating that SNX27 haploinsufficiency suppresses the proliferation of cells in injured spinal cords. Furthermore, fewer cells positive for both Iba1 and Ki67 was observed in the lesion site of *Snx27*^+/−^ mice following SCI, compared with those in *Snx27*^+/+^ littermates (Figure 7B). Therefore, SNX27 haploinsufficiency suppresses proliferation of macrophage/microglia following SCI.

## Discussion

SNX27, an endosome-associated cargo adaptor, is involved in developmental and neurological diseases, such as Down syndrome, Alzheimer's disease, infantile myoclonic epilepsy, hydrocephalus, and neuropathic pain (24–26, 30). Previous reports found that SNX27 is involved in neuropathic pain in a mouse spinal nerve ligation model (30). However, before our study, the physiological function of SNX27 in SCI has not been investigated. For the first time, we determined the roles of SNX27 in SCI, which will extend our understanding the functional regulation of SNX27 in spinal cord injury.

In this study, we found that SNX27 expression starts to increase on day 3 and reached the plateau on day 7 after SCI (Figure 1B). Moreover, we found that the expression of SNX27 was markedly upregulated around the lesion site where the necrotic neurons and activated microglia/macrophage emerged after spinal cord injury (Figure 1C). These results suggest that SNX27 is associated with neuronal death and neuroinflammation, and may function in the early stage of SCI. Furthermore, we investigate the physiological function of SNX27 in SCI using *Snx27*^+/−^ mice since that loss of Snx27 results in severe neuronal death and early lethality in *Snx27*^−^^/−^ mice. We have found that SNX27 haploinsufficiency elevated corticospinal axon regeneration from the caudal area to the lesion area (Figure 3) and improved functional motor recovery after SCI (Figure 2), suggesting that SNX27 may have an effect on functional recovery after spinal cord injury.

Previous studies have shown that down-regulation of NMDA receptors, such as GluN1 and GluN2B, contributes to reduction of neuronal death due to CNS injury, but is accompanied by side-effects (7, 8). Moreover, SNX27-deficiency decreased glutamate receptor recycling to the post-synaptic surface (24). Therefore, we put forward the hypothesis that deficiency of SNX27 might protect neurons from Glutamate-induced excitotoxicity in SCI damage by reducing the surface expression of NMDA receptors. We found that SNX27 haploinsufficiency significantly promoted neuronal survival adjacent to the lesion site and reduced expression of apoptotic cleaved caspase-3 after SCI (Figure 4). This indicates SNX27 haploinsufficiency protects neurons from SCI-induced apoptotic cell death and possibly by blocking NMDA receptor activation.

A sequential inflammatory cascade is initiated after spinal cord injury (40), microglia and astrocyte, two types of glial cells reside in the spinal cord are likely contributors. They can be activated to various degrees after spinal cord injury (41, 42). Although there are different functional states of macrophage/microglia activated after SCI, treatments aimed at anti-inflammatory pathways have been a mainstay of pre-clinical SCI research for many years (43). Macrophage/microglia that infiltrate in the injured area can secrete inflammatory factors such as TNFα, IL-1, IL-6, aggravating neuronal damage and cavity formation, while suppressing neurogenesis and axonal regeneration (36, 44). In the days following the initial damage to the spinal cord, secondary damage continues in the tissue surrounding the original site of injury, spinal cavity (GFAP-negative) formed and enlarged gradually with reactive astrocytes surrounded it, which are exacerbating neurological defects (41, 45). Proliferation of astrocytes at the early stage of SCI can limit migration of the immune cells toward the injured spinal cord (3). However, over time, astrocytes produce extensive glial scarring, which restricts the regeneration and extension of axons (3). Consistent with previous reports, we found that there were more macrophage/microglia infiltrating in the spinal cord of *Snx27*^+/+^ mice compared with *Snx27*^+/−^ mice (Figure 6). Moreover, the lesion site (GFAP-negative) was significantly smaller in *Snx27*^+/−^ mice, although there was no difference in astrogliosis between *Snx27*^+/+^ and *Snx27*^+/−^ mice (Figure 5). SNX27 haploinsufficiency reduced the number of infiltrating Iba1^+^/Ki67^+^ cells (newborn microglia/macrophage) in the lesion sites after SCI (Figure 7). Thus, SNX27 haploinsufficiency suppresses the inflammatory response by inhibiting the macrophage/microglia proliferation after SCI, but SNX27 has no effect on astrogliosis in this SCI model.

## Conclusion

In summary, our findings demonstrate a pathological function of SNX27 in spinal cord injury by increasing neuroinflammation and neuronal apoptotic death. The details of the underlying mechanism deserve further scrutiny.

## Ethics Statement

All animal procedures were in strict accordance with the National Institutes of Health's Guidelines for Care and were approved by the Animal Ethics Committee of Xiamen University.

## Author Contributions

YZ, XW, and BL conceived the study. YZ designed and performed the experiments. TG, ShW, SoW, XL, JW, and ZC provided additional advice in experimental design and execution. HX provided discussion. YZ, XW, and BL wrote the manuscript. NW and QZ edited the manuscript. XW and BL supervised the project.

### Conflict of interest statement

The authors declare that the research was conducted in the absence of any commercial or financial relationships that could be construed as a potential conflict of interest.

## References

[1] Fehlings M Singh A Tetreault L Kalsi-Ryan S Nouri A. Global prevalence and incidence of traumatic spinal cord injury. Clin Epidemiol. (2014) 6:309–31. 10.2147/CLEP.S68889 25278785 PMC4179833

[2] Manzhulo O Tyrtyshnaia A Kipryushina Y Dyuizen I Manzhulo I. Docosahexaenoic acid induces changes in microglia/macrophage polarization after spinal cord injury in rats. Acta Histochem. (2018) 120:741–7. 10.1016/j.acthis.2018.08.005 30170694

[3] Gomes-Leal W Corkill DJ Freire MA Picanco-Diniz CW Perry VH. Astrocytosis, microglia activation, oligodendrocyte degeneration, and pyknosis following acute spinal cord injury. Exp Neurol. (2004) 190:456–67. 10.1016/j.expneurol.2004.06.028 15530884

[4] Jia YF Gao HL Ma LJ Li J. Effect of nimodipine on rat spinal cord injury. Genet Mol Res. (2015) 14:1269–76. 10.4238/2015.February.13.5 25730065

[5] Li Y Gu R Zhu Q Liu J. Changes of spinal edema and expression of aquaporin 4 in methylprednisolone-treated rats with spinal cord injury. Ann Clin Lab Sci. (2018) 48:453–9. 30143486

[6] Becker D Sadowsky CL McDonald JW. Restoring function after spinal cord injury. Neurologist (2003) 9:1–15. 10.1097/01.nrl.0000038587.58012.05 12801427

[7] Huang M Cheng G Tan H Qin R Zou Y Wang Y. Capsaicin protects cortical neurons against ischemia/reperfusion injury via down-regulating NMDA receptors. Exp Neurol. (2017) 295:66–76. 10.1016/j.expneurol.2017.05.001 28479337 PMC5991616

[8] Zhang Z Liu J Fan C Mao L Xie R Wang S. The GluN1/GluN2B NMDA receptor and metabotropic glutamate receptor 1 negative allosteric modulator has enhanced neuroprotection in a rat subarachnoid hemorrhage model. Exp Neurol. (2017) 301(Pt A):13–25. 10.1016/j.expneurol.2017.12.005 29258835

[9] Horn KP Busch SA Hawthorne AL van Rooijen N Silver J. Another barrier to regeneration in the CNS: activated macrophages induce extensive retraction of dystrophic axons through direct physical interactions. J Neurosci. (2008) 28:9330–41. 10.1523/JNEUROSCI.2488-08.2008 18799667 PMC2567141

[10] Beattie MS. Inflammation and apoptosis: linked therapeutic targets in spinal cord injury. Trends Mol Med. (2004) 10:580–3. 10.1016/j.molmed.2004.10.006 15567326

[11] Slaets H Nelissen S Janssens K Vidal PM Lemmens E Stinissen P. Oncostatin M reduces lesion size and promotes functional recovery and neurite outgrowth after spinal cord injury. Mol Neurobiol. (2014) 50:1142–51. 10.1007/s12035-014-8795-5 24996996

[12] Bethea JR. Spinal cord injury-induced inflammation: a dual-edged sword. Progr Brain Res. (2000) 128:33–42. 10.1016/S0079-6123(00)28005-9 11105667

[13] Donnelly DJ Longbrake EE Shawler TM Kigerl KA Lai W Tovar CA. Deficient CX3CR1 signaling promotes recovery after mouse spinal cord injury by limiting the recruitment and activation of Ly6Clo/iNOS+ macrophages. J Neurosci. (2011) 31:9910–22. 10.1523/JNEUROSCI.2114-11.2011 21734283 PMC3139517

[14] Courtine G van den Brand R Musienko P. Spinal cord injury: time to move. Lancet (2011) 377:1896–8. 10.1016/S0140-6736(11)60711-3 21601272

[15] Gris D Marsh DR Oatway MA Chen Y Hamilton EF Dekaban GA. Transient blockade of the CD11d/CD18 integrin reduces secondary damage after spinal cord injury, improving sensory, autonomic, and motor function. J Neurosci. (2004) 24:4043–51. 10.1523/JNEUROSCI.5343-03.2004 15102919 PMC6729422

[16] Popovich PG Guan Z Wei P Huitinga I van Rooijen N Stokes BT. Depletion of hematogenous macrophages promotes partial hindlimb recovery and neuroanatomical repair after experimental spinal cord injury. Exp Neurol. (1999) 158:351–65. 10.1006/exnr.1999.7118 10415142

[17] Stirling DP Khodarahmi K Liu J McPhail LT McBride CB Steeves JD. Minocycline treatment reduces delayed oligodendrocyte death, attenuates axonal dieback, and improves functional outcome after spinal cord injury. J Neurosci. (2004) 24:2182–90. 10.1523/JNEUROSCI.5275-03.2004 14999069 PMC6730425

[18] Mallory GW Grahn PJ Hachmann JT Lujan JL Lee KH. Optical Stimulation for Restoration of Motor Function After Spinal Cord Injury. Mayo Clin Proc. (2015) 90:300–7. 10.1016/j.mayocp.2014.12.004 25659246 PMC4339262

[19] Estrada V Muller HW Spinal cord injury - there is not just one way of treating it. F1000Prime Rep. (2014) 6:84 10.12703/P6-84 25343041 PMC4166939

[20] Wang J Pearse DD. Therapeutic hypothermia in spinal cord injury: the status of its use and open questions. Int J Mol Sci. (2015) 16:16848–79. 10.3390/ijms160816848 26213924 PMC4581174

[21] de Rivero Vaccari JP Dietrich WD Keane RW. Therapeutics targeting the inflammasome after central nervous system injury. Transl Res. (2016) 167:35–45. 10.1016/j.trsl.2015.05.003 26024799 PMC4643411

[22] Gallon M Clairfeuille T Collins BM Cullen PJ. A unique PDZ domain and arrestin-like fold interaction reveals mechanistic details of endocytic recycling by SNX27-retromer. Mol Biol Cell (2014) 111:E3604–13. 10.1073/pnas.1410552111 25136126 PMC4156734

[23] Cai L Loo LS Atlashkin V Hanson BJ Hong W. Deficiency of sorting nexin 27 (SNX27) leads to growth retardation and elevated levels of N-methyl-D-aspartate receptor 2C (NR2C). Mol Cell Biol. (2011) 31:1734–47. 10.1128/MCB.01044-10 21300787 PMC3126336

[24] Wang X Zhao Y Zhang X Badie H Zhou Y Mu Y. Loss of sorting nexin 27 contributes to excitatory synaptic dysfunction by modulating glutamate receptor recycling in Down's syndrome. Nat Med. (2013) 19:473–80. 10.1038/nm.3117 23524343 PMC3911880

[25] Wang X Huang T Zhao Y Zheng Q Thompson RC Bu G. Sorting nexin 27 regulates Abeta production through modulating gamma-secretase activity. Cell Rep. (2014) 9:1023–33. 10.1016/j.celrep.2014.09.037 25437557 PMC4328673

[26] Huang TY Zhao Y Li X Wang X Tseng IC Thompson R. SNX27 and SORLA interact to reduce amyloidogenic subcellular distribution and processing of amyloid precursor protein. J Neurosci. (2016) 36:7996–8011. 10.1523/JNEUROSCI.0206-16.2016 27466343 PMC4961782

[27] Damseh N Danson CM Al-Ashhab M Abu-Libdeh B Gallon M Sharma K. A defect in the retromer accessory protein, SNX27, manifests by infantile myoclonic epilepsy and neurodegeneration. Neurogenetics (2015) 16:215–21. 10.1007/s10048-015-0446-0 25894286 PMC4962907

[28] Wang X Zhou Y Wang J Tseng IC Huang T Zhao Y. SNX27 Deletion causes hydrocephalus by impairing ependymal cell differentiation and ciliogenesis. J Neurosci. (2016) 36:12586–97. 10.1523/JNEUROSCI.1620-16.2016 27974614 PMC5157104

[29] Munoz MB Slesinger PA. Sorting nexin 27 regulation of G protein-gated inwardly rectifying K(+) channels attenuates *in vivo* cocaine response. Neuron (2014) 82:659–69. 10.1016/j.neuron.2014.03.011 24811384 PMC4141045

[30] Lin TB Lai CY Hsieh MC Wang HH Cheng JK Chau YP. VPS26A-SNX27 Interaction-dependent mGluR5 recycling in dorsal horn neurons mediates neuropathic pain in rats. J Neurosci. (2015) 35:14943–55. 10.1523/JNEUROSCI.2587-15.2015 26538661 PMC6605230

[31] Cheriyan T Ryan DJ Weinreb JH Cheriyan J Paul JC Lafage V. Spinal cord injury models: a review. Spinal Cord (2014) 52:588–95. 10.1038/sc.2014.91 24912546

[32] Mostacada K Oliveira FL Villa-Verde DM Martinez AM. Lack of galectin-3 improves the functional outcome and tissue sparing by modulating inflammatory response after a compressive spinal cord injury. Exp Neurol. (2015) 271:390–400. 10.1016/j.expneurol.2015.07.006 26183316

[33] Wang SM Hsu JC Ko CY Chiu NE Kan WM Lai MD. Astrocytic CCAAT/enhancer-binding protein delta contributes to glial scar formation and impairs functional recovery after spinal cord injury. Mol Neurobiol. (2016) 53:5912–27. 10.1007/s12035-015-9486-6 26510742 PMC5085997

[34] Danilov CA Steward O. Conditional genetic deletion of PTEN after a spinal cord injury enhances regenerative growth of CST axons and motor function recovery in mice. Exp Neurol. (2015) 266:147–60. 10.1016/j.expneurol.2015.02.012 25704959 PMC4382431

[35] Liu K Lu Y Lee JK Samara R Willenberg R Sears-Kraxberger I. PTEN deletion enhances the regenerative ability of adult corticospinal neurons. Nature Neurosci. (2010) 13:1075–81. 10.1038/nn.2603 20694004 PMC2928871

[36] Du K Zheng S Zhang Q Li S Gao X Wang J. Pten deletion promotes regrowth of corticospinal tract axons 1 year after spinal cord injury. J Neurosci. (2015) 35:9754–63. 10.1523/JNEUROSCI.3637-14.2015 26134657 PMC6605149

[37] Lee JY Chung H Yoo YS Oh YJ Oh TH Park S. Inhibition of apoptotic cell death by ghrelin improves functional recovery after spinal cord injury. Endocrinology (2010) 151:3815–26. 10.1210/en.2009-1416 20444938

[38] Lee JY Kang SR Yune TY. Fluoxetine prevents oligodendrocyte cell death by inhibiting microglia activation after spinal cord injury. J Neurotrauma (2015) 32:633–44. 10.1089/neu.2014.3527 25366938 PMC4410451

[39] Scholzen T Gerdes J. The Ki-67 protein: from the known and the unknown. J Cell Physiol. (2000) 182:311–22. 10.1002/(SICI)1097-4652(200003)182:3<311::AID-JCP1>3.0.CO;2-9 10653597

[40] Gensel JC Zhang B. Macrophage activation and its role in repair and pathology after spinal cord injury. Brain Res. (2015) 1619:1–11. 10.1016/j.brainres.2014.12.045 25578260

[41] Fan H Zhang K Shan L Kuang F Chen K Zhu K. Reactive astrocytes undergo M1 microglia/macrohpages-induced necroptosis in spinal cord injury. Mol Neurodegener. (2016) 11:14. 10.1186/s13024-016-0081-8 26842216 PMC4740993

[42] Gaudet AD Mandrekar-Colucci S Hall JCE Sweet DR Schmitt PJ Xu X. miR-155 deletion in mice overcomes neuron-intrinsic and neuron-extrinsic barriers to spinal cord repair. J Neurosci. (2016) 36:8516–32. 10.1523/JNEUROSCI.0735-16.2016 27511021 PMC4978808

[43] Squair JW Ruiz I Phillips AA Zheng MMZ Sarafis ZK Sachdeva R. Minocycline reduces the severity of autonomic dysreflexia after experimental spinal cord injury. J Neurotrauma (2018). [Epub ahead of print] 10.1089/neu.2018.5703. 30113266

[44] Lopes RS Cardoso MM Sampaio AO Barbosa MS Jr Souza CC MC DAS. Indomethacin treatment reduces microglia activation and increases numbers of neuroblasts in the subventricular zone and ischaemic striatum after focal ischaemia. J Biosci. (2016) 41:381–94. 10.1007/s12038-016-9621-1 27581930

[45] Boato F Hendrix S Huelsenbeck SC Hofmann F Grosse G Djalali S. C3 peptide enhances recovery from spinal cord injury by improved regenerative growth of descending fiber tracts. J Cell Sci. (2010) 123(Pt 10):1652–62. 10.1242/jcs.066050 20406886

